# Connecto-informatics at the mesoscale: current advances in image processing and analysis for mapping the brain connectivity

**DOI:** 10.1186/s40708-024-00228-9

**Published:** 2024-06-04

**Authors:** Yoon Kyoung Choi, Linqing Feng, Won-Ki Jeong, Jinhyun Kim

**Affiliations:** 1 Brain Science Institute, Korea Institute of Science and Technology (KIST), Seoul, South Korea; 2 Department of Computer Science and Engineering, Korea University, Seoul, South Korea; 3 Zhejiang Lab, Hangzhou, China; 4 KIST-SKKU Brain Research Center, SKKU Institute for Convergence, Sungkyunkwan University, Suwon, South Korea

**Keywords:** Image processing, Brain mapping, Atlas registration, Atlas segmentation, Mesoscale connectivity, Neuron reconstruction

## Abstract

Mapping neural connections within the brain has been a fundamental goal in neuroscience to understand better its functions and changes that follow aging and diseases. Developments in imaging technology, such as microscopy and labeling tools, have allowed researchers to visualize this connectivity through high-resolution brain-wide imaging. With this, image processing and analysis have become more crucial. However, despite the wealth of neural images generated, access to an integrated image processing and analysis pipeline to process these data is challenging due to scattered information on available tools and methods. To map the neural connections, registration to atlases and feature extraction through segmentation and signal detection are necessary. In this review, our goal is to provide an updated overview of recent advances in these image-processing methods, with a particular focus on fluorescent images of the mouse brain. Our goal is to outline a pathway toward an integrated image-processing pipeline tailored for connecto-informatics. An integrated workflow of these image processing will facilitate researchers’ approach to mapping brain connectivity to better understand complex brain networks and their underlying brain functions. By highlighting the image-processing tools available for fluroscent imaging of the mouse brain, this review will contribute to a deeper grasp of connecto-informatics, paving the way for better comprehension of brain connectivity and its implications.

## Introduction

The brain consists of a complex network, intricately interconnected through countless neurons connecting various brain regions. These networks form the foundation for critical brain functions such as movement, social interaction, memory formation, decision-making, and perception. By investigating the structural and functional properties of neural circuits, researchers seek to understand and uncover the fundamental principles of information processing in the brain. A key focus in neuroscience has been to map neural circuits, which involves visualizing and characterizing the connections between neurons to deepen our understanding of brain organization. This mapping is crucial for grasping normal brain functions and addressing related disorders.

Over the past decade, significant progress has been made in mapping the connectivity of the brain at the mesoscale level, which encompasses intermediate scales between individual neurons and large brain regions [1–5]. Although various connectivity studies utilize numerous animal models [1, 3, 6–9], connectome research primarily leverages mouse models due to their prevalence. This review will focus on the mouse brain, examining mesoscale connectivity through fluorescent imaging. This mesoscale connectivity mapping provides insights into the structural and functional relationships between brain regions, shedding light on information flow, neural circuits, and their contributions to overall brain functionality.

The term connecto-informatics, first introduced in a study that investigated the circuit- and cellular-level connectivity of the STN-GPe [10], is analogous to neuroinformatics but focuses specifically on extracting and analyzing information about neural connectivity. In the context of connecto-informatics, researches employ various imaging and computational techniques, data analysis and modeling to deepen our understanding of brain structure and function through brain circuit connectivity. Recently, advances in neural labeling, tissue clearing, and imaging methods – such as mGRASP, CLARITY, iDISCO, MOST, fMOST, and ExM – have significantly accelerated neural circuit mapping efforts [11–20]. The datasets primarily used in connecto-informatics are fluorescence-based, and there has been a high throughput of fluorescence imaging [21]. However, fluorescence imaging datasets often contain discrepancies due to biological variations such as brain size differences among animals, inevitable damages from histological sample processing, and technical problems like artifacts and optical aberrations [22, 23]. These can lead to signal loss and image distortion, underscoring the critical need for sophisticated image processing tools and methodologies in mesoscopic connectivity mapping.

**Fig. 1:**
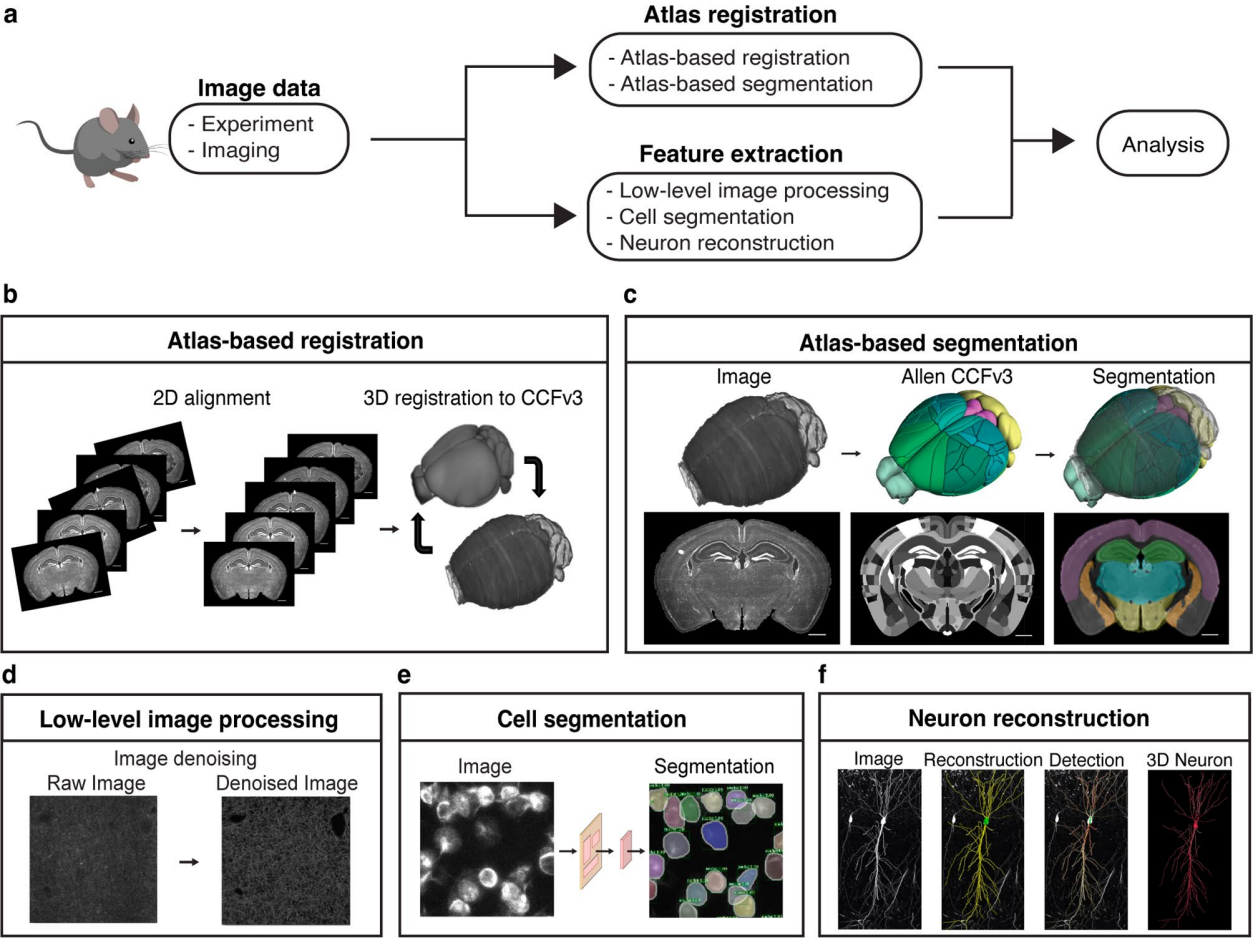
Workflow of image processing for connecto-informatics at mesoscale. (**a**) Schematics diagram of key image processing steps for neural data obtained from imaging. (**b**) Whole brain images obtained in 2D are aligned into a 3D stack and is registered to the Allen CCFv3 [26]. (**c**) The registered neural images are segmented using the annotated Allen CCFv3. (**d**) Noisy images are processed using denoising algorithm to remove unwanted artifacts that can arise from numerous factors [65]. (**e**) Cell segmentation using CNN allow automatic detection and segmentation of cells in neural images, allowing cellular level connectivity analysis [97]. (**f**) 3D reconstruction of neuron using neuTube 1.0 shows synaptic connectivity of hippocampal region with mGRASP labeled synapses [110]. All scale bar represents 1000μm

By extracting information through analysis of neural connectivity mapping from neural images obtained through various microscopies and using various emerging image processing tools, extraction, analysis, and interpretation of complex brain connectivity data is possible. The image processing pipeline in connecto-informatics begins with the vital step of aligning neural images to a standardized template atlas. This is followed by segmentation of specific brain regions or structures, essential for isolating areas of interest for detailed analysis. To improve image quality and clarity, the pipeline incorporates low-level techniques such as denoising and super-resolution, enabling the visualization of finer structural details [57–62, 71–77]. Advanced procedures, including cell segmentation and neuronal morphology reconstruction [91–110], are also employed to comprehend the intricate connectivity and dynamics of neural circuits at the mesoscopic scale. The ultimate goal of these image processing steps is to accurately map and analyze neural circuits, providing insights into the complex networks of connectivity and interactions that underpin various brain functions (Fig. 1a).

Despite advancements, the availability and integration of image-processing resources remain scattered, posing challenges in their effective utilization. The substantial data volume generated by sophisticated imaging techniques demands significant computational resources and processing time. Variability in biological samples and imaging conditions adds complexity, often requiring customized approaches and manual intervention, which impedes the development of streamlined, automated workflows. Moreover, integrating and analyzing diverse data types to map neural circuits efficiently remains a formidable challenge, highlighting a gap between the capabilities of current tools and research needs.

In this updated review, we delve into the advancements in image processing tools for mapping mesoscopic brain connectivity, addressing the challenges encountered and introducing tools to overcome them.

- Mapping brain connectivity through atlas-based registration.
  - Types of Brain Atlases.
  - Atlas-based Registration and Segmentation.
  - Atlas-based registration and segmentation open-source tools.
  - Deep learning-based atlas-based registration and segmentation tools.
- Mapping brain connectivity through feature extraction.
  - Low-level image processing
    - Image Denoising.
    - Image super-resolution.
  - Cell Segmentation.
  - Neurononal morphology reconstruction.

Starting with a brief introduction to the importance and significance of each image processing step, we discuss latest advancements in tools and methods tailored for analyzing neural images in the context of connecto-informatics at the mesoscale (Abbreviations listed in Table 1). This review aims to provide insights into the current state of image processing techniques and their pivotal role in advancing our understanding of brain connectivity.

**Table 1:** List of abbreviations used in this review

Abbreviation	Full name	Reference
Allen CCFv3	Allen Common Coordinate Framework v3	[26]
ANTs	Advanced Normalization Tools	[38]
BIRDS	Bi-channel image registration and deep-learning segmentation	[53]
CARE	Content-aware image restoration	[59]
CNN	Convolution neural network	[49, 52, 55–58, 61, 77, 99, 105, 106, 139–141]
DNN	Deep neural network	[53, 57, 78, 98, 100]
ExM	Expansion Microscopy	[11]
FNT	Fast Neurite Tracer	[120–122]
GAN	Generative adversarial networks	[74, 75, 79, 142]
ITK	Insight toolkit	[37]
mGRASP	mammalian GFP reconstitution across synaptic partners	[12]
MIRACL	Multimodal Image Registration And Connectivity anaLysis	[45]
MOST	Micro-optical sectioning tomography	[20, 117, 120]
mPFC	Medial prefrontal cortex	[120]
SNR	Signal-to-noise ratio	[54]
STN	Subthalamic nucleus	[10]
STPT	Serial two-photon tomography	[26, 87]
Vaa3D	3D Visualization-Assisted Analysis	[113]

## Mapping brain connectivity through atlas-based registration

Many connectivity datasets are derived from various resolutions and imaging modalities, necessitating the crucial first step of registering images onto a standardized reference framework. This alignment enables comparative analyses across different experiments, datasets, and subjects, providing valuable information about neuronal structures and functions. Moreover, registering images to a common coordinate space facilitates the annotation of brain regions and is fundamental for qualitative and quantitative assessments. This allows for more precise comparisons and analyses of specific regions of interest. Systematic image processing, including steps like registration and segmentation against reference atlases, is essential for accurately mapping neural connectivity.

### Types of brain atlases

A brain atlas is a comprehensive and detailed map illustrating the brain’s anatomical structures and functional organization. In connecto-informatics, whole-brain atlases provide a spatial framework for analyzing whole-brain images. The Franklin-Paxinos atlas [24] and the Allen reference atlas [25] are among the most widely used for mouse brains. However, these are 2D reference atlases, primarily derived from Nissl and acetylcholine esterase antibody staining in histological sections. Although reference atlases such as the Franklin-Paxinos or Allen have assisted researchers in locating and annotating brain regions of interest, their 2D nature limits their effectiveness and application.

The shift to 3D brain atlas, like the Allen Common Coordinate Framework v3 (CCFv3) offers several advantages including spatial accuracy, depth visualization, cross-sectional view of volumetric data, and navigational aids. The Allen CCFv3 is a 3D whole-brain mouse atlas available through the Allen Institute for Brain Science (https://mouse.brain-map.org). It was created interpolating serial two-photon tomography (STPT) images from 1675 adult mice and features 658 delineated brain regions. This atlas integrates data from immunohistochemistry, transgene expression, in situ hybridization, and anterograde tracer connectivity data [26].

**Fig. 2:**
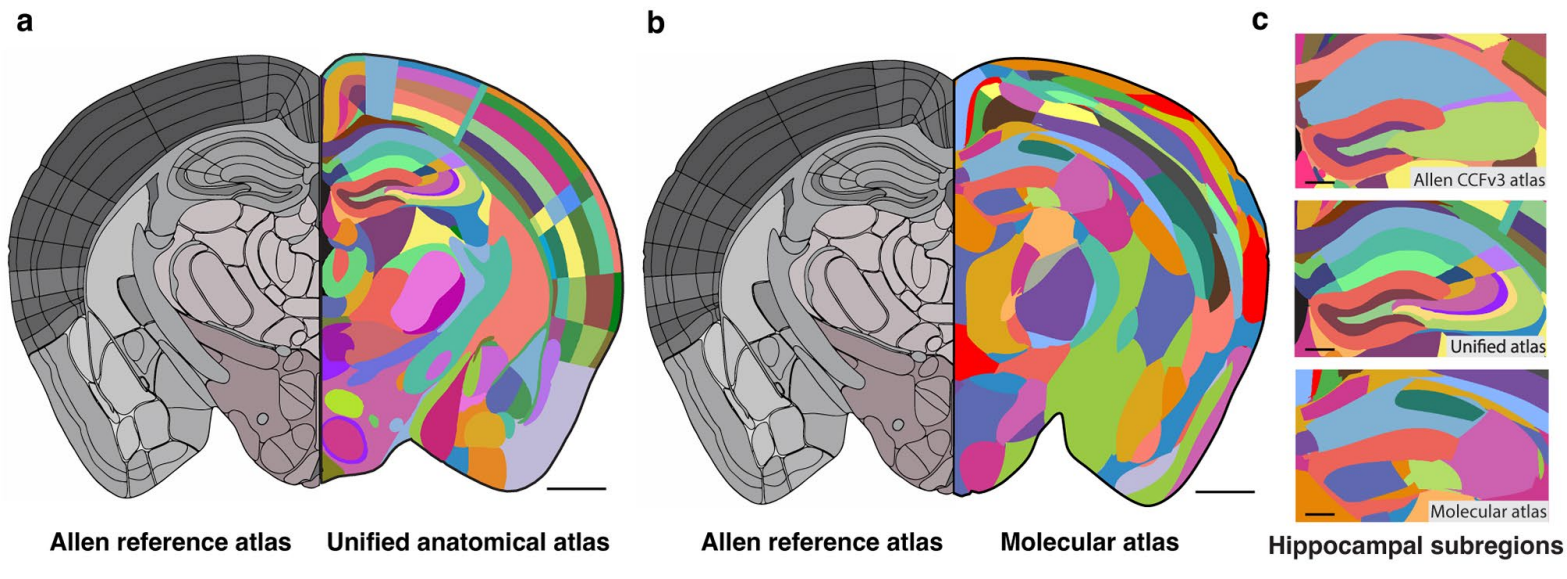
Comparison of mouse brain atlases. Rebuilt illustration using publicly available atlases for comparison between the enhanced and unified anatomical atlas, and the molecular atlas of the mouse brain combined. (**a**) The left hemisphere is the Allen reference atlas [26] and the right hemisphere is the enhance and unified mouse brain atlas that combines labels from the Franklin-Paxinos atlas and the common coordinate framework from the Allen Institute to create a unified mouse brain atlas [27]. (**b**) The left hemisphere is the Allen reference atlas and the right hemisphere is the molecular atlas of the adult mouse brain that shows anatomical divisions based molecular composition [29]. (**c**) Comparison of the hippocampus region delineation between mouse brain atlases. Scale bars for (**a**-**b**) represents 1000μm; scale bar for (**c**) represents 500μm

To enhance anatomical delineation, the 2D segmentation labels from the Franklin-Paxinos atlas have been merged onto the Allen CCFv3, creating an enhanced and unified mouse brain atlas [27] (Fig. 2a). Though this atlas is based on the Allen CCFv3, additional anatomical regions were further segmented by combining data from cell type-specific transgenic mice and MRI. Other mouse brain 3D atlases were also developed using unsupervised classification single-cell RNA profiles to define anatomical divisions based molecular composition (Fig. 2b). The gene expression signatures are obtained using spatial transcriptomics of mRNAs [28, 29]. These atlases help in identifying distinct subregions by segmenting the hippocampal subfields into sublayers or revealing unique patterns at the dorsoventral borders in the hippocampal subfields (Fig. 2c). Using the Allen CCFv3, these atlases have added important information about region segmentation, gene, and cell expressions, allowing researchers to compare significant results across experiments onto a common reference framework.

Creating an atlas that accurately delineates regions to match biological features remains challenging. Efforts are also underway to develop a developmental mouse brain atlas covering various age points, which is critical for understanding growth and development stages [30–32]. Given that providing a generalized adult brain atlas is a challenge, creating a lifespan atlas is an even more significant challenge. Currently, easy online access to comprehensive 3D atlas similar to those available of adult mice is not yet reality for developmental stages. However, the establishment of a standardized developing mouse atlas would mark a significant advancement. It would provide a generalized framework for studying the developing mouse brain and analyzing the connecto-informatics of brain circuits throughout various stages of development.

### Atlas-based registration and segmentation

From connecto-informatics analysis, integrating images into a reference space is crucial for extracting neural information, requiring a registration process. Image registration involves spatially aligning two images from various modalities to identify or correlate changes in structure or function [33] (Fig. 1b). Specifically, this process entails merging a neural image with a reference image – typically a corresponding 2D section from an atlas – for detailed analysis of neural circuit. There are two primary methods for this integration. One approach maps the reference image onto a neural image, maintaining the integrity of the neural image without distortion. Alternatively, the image data can be transformed to fit the reference space, which, while potentially distorting the original image data, facilitates comparison across different datasets and experiments within the same reference framework.

Image registration is a critical step in neural analysis, typically performed using transformations provided by open-source libraries [34, 35], such as NiftyReg [36], Elastix [37], and ANTs [38], which are widely recognized for their effectiveness. Elastix and ANTs, built on the ITK framework, employ both linear and non-linear transformations to align sample data with reference images through deformation processes. This precise alignment is crucial for the subsequent step of brain region segmentation, as the accuracy of segmentation directly depends on how well the brain and atlas have been registered. This segmentation step usually occurs after the reference image has been aligned with the data image (Fig. 1c). Even though these open-source libraries are readily available, they require a degree of computational expertise, posing a barrier for many biologists. Additionally, the lack of standardized methods implementing data complicated the use of these tools on diverse datasets, presenting ongoing challenges for researchers in the field.

Atlases serve as essential backbone for connecto-informatics, with registration and segmentation of brain regions heavily dependent on them. However, inconsistencies in the boundaries of each segmented region across different atlases can significantly impact analysis outcomes. Therefore, selecting the appropriate atlases is crucial for ensuring reliable results, and the use of uniform atlases could facilitate the establishment of a standardized research pipeline.

Efforts are underway to address these challenges and improve the accessibility for researchers. One notable attempt is the BrainGlobe [39], a platform that consolidates available atlases to offer a common interface processing data across various model organisms. While significant progress has been made in creating more accurate and accessible atlases, the need for standardized and precise reference atlases remains paramount. These atlases not only support atlas based image processing but also enhance the integration and combination of diverse datasets from different research projects, fostering collaborative efforts in brain connectivity mapping.

### Atlas-based registration and segmentation open-source tools

The advancement of atlas-based registration and segmentation tools, alongside with the development of standardized brain atlases, has significantly advanced neuroscience image processing, particularly in mapping mesoscale neural circuits. These tools, by simplifying the alignment of neural images to brain atlases, address critical challenges like the need for high-level computational resources and expertise. They also help mitigate issues associated with damaged or incomplete datasets.

One of the recent developments are tools designed for 2D and 3D image processing tasks. Software like WholeBrain [40] and Neuroinfo [41] has emerged to offer semi-automatic solutions for 2D registration and segmentation, utilizing advanced algorithms and integrating the comprehensive Allen CCFv3 brain atlas. These tools are specifically engineered to simplify the initial stages of image processing, enabling researchers to accurately align experimental data with reference spaces and automatically annotate critical regions based on the atlas. This process is significantly facilitated by the software’s capability to automatically register image data to the reference slice once the researcher identifies the corresponding region in the 2D section image. However, these tools are not without limitations; they can be time-consuming to use and may not offer the necessary flexibility for handling various image modalities, highlighting a trade-off between automation and adaptability.

Recognizing the time-intensive nature of manual 2D registration, QuickNII offers an advanced semi-automatic approach that significantly reduces the effort required to register serial section data to a 3D reference atlas [42]. By applying affine spatial transformations, QuickNII efficiently aligns each section across the entire series, alleviating one of the most laborious aspects of neural image processing. Similarly, FASTMAP, a plugin for ImageJ, generates custom mouse brain atlas plates [43]. This feature address the unique requirements of diverse experimental setups, enhancing the tool’s utility and flexibility in registration tasks.

Transitioning from 2D to 3D, tools like aMAP, MIRACL, and MagellanMapper are each designed to address the complexities of 3D registration and segmentation. aMAP, leveraging the NiftyReg framework, offers a validated approach that aligns with expert manual segmentation for fluorescent mouse brain images [44]. This validation ensures that researchers can rely on aMAP for accurate 3D analysis. MIRACL [45] and MagellanMapper [46] further extend the capabilities of 3D image processing, implementing fully automated registration pipelines tailored for cleared brain images and diffusion MRI data. By utilizing frameworks like ANTs and Elastix, these tools not only automate the processing of high-resolution data but also assure precision in aligning and segmenting images within the 3D neural features.

The transition from manual registration libraries to sophisticated, user-friendly software tools in neuroscience reflects ongoing efforts to address image processing challenges. While these tools have significantly streamlined processing and reduced manual intervention, they continue to evolve to meet the increasing complexity of imaging data and analysis demands. Despite these advancements, practical challenges persist, particularly with atlas-based registration and segmentation. Variability among individual brains can lead to registration errors, and the existing atlases may not capture all anatomical variations needed for specific research, underscoring the limitations in completeness and specificity. Consequently, expert judgment remains crucial in interpreting and correcting misalignments, ensuring accurate segmentation and integration. This blend of technological advancement and the need for skilled human oversight highlights the enduring necessity for expert involvement in refining and utilizing these advanced tools.

### Deep learning-based atlas-based registration and segmentation tools

With the rapid progress in artificial intelligence (AI), significant efforts have been made towards developing deep learning-based tools for automatic registration and segmentation, aiming to ease the bottleneck caused by the vast volumes of image data generated. DeepSlice, an automated registration library, aligns and registers mouse brain histology data to the Allen CCFv3 from the Allen Brain Institute [47]. This tool uses estimated Euclidean data to provide a standardized and simplified registration process. Additionally, Mesonet facilitates automatic mouse brain segmentation by utilizing landmarks on brain image to automate segmentation according to the atlas [48]. Furthermore, DeepMapi, a fully automated registration method for mesoscopic optical brain images, uses convolution neural network (CNN) to predict deformation field to align mesoscopic images with the atlas, demonstrating how deep learning can be used to streamline these processes [49].

Another notable software is mBrainAligner, an open-source software for cross-modal registration that employs deep neural network (DNN) to align whole mouse brain with the the standard Allen CCFv3 atlas [50]. mBrainAligner has shown more accurate segmentation results compared to the tools mentioned above. The implementation of deep learning in such software not only accelerates processing but also achieves results comparable to manual registration and segmentation, thereby ensuring high accuracy. Additionally, D-LMBmap has been developed as a fully automated, deep learning-based end-to-end package for comprehensive profiling of neural circuity across the entire brain [51]. This tool provides an integrated workflow that encompasses whole-brain registration, region segmentation, and axon segmentation, facilitating brain circuit profiling with minimum manual input. Although currently limited to light sheet fluorescent microscopy, D-LMBmap features a novel method of registration and segmentation with a user-friendly graphical interface. Once validated on high-resolution images, it will be a powerful tool with competitiveness comparable to other already available software. These developments in deep learning-based software allow high throughput automatic registration without manual intervention. This capability allows for the rapid precise analysis of vast dataset generated by advanced imaging technology.

Deep learning-based registration and segmentation tools like DeepBrainSeg and BIRDS have not only streamline the processes of registration and segmentation but have also addressed more complex challenges inherent in neural data processing. DeepBrainSeg is an automated brain region segmentation tool for micro-optical images that employs dual-pathway CNN to capture both local details and broader contextual information across various scales [52]. This approach significantly enhances the accurate segmentation of brain regions, even in noisy datasets, through sophisticated image registration and the application of domain-specific constraints.

BIRDS, a Fiji plugin software, extends the utility of deep learning by offering an open-source algorithm that can be implemented on various image modalities, allowing easy access and usability to many users [53]. In addition to providing automatic registration and segmentation, BIRDS offers a deep learning-based direct-inference segmentation on incomplete datasets, such as irregularly or partially cut brain sections or hemispheres. These types of datasets often present considerable challenges due to their lack of comprehensive morphological information, making traditional segmentation based on standard atlases like the Allen brain atlas difficult. By integrating DNN, BIRDS effectively segments these partial images.

The continued development of deep learning-based, open-source tools for registration and segmentation represents a significant advancement in preprocessing neural images. These tools have transformed the image processing procedure, making it more convenient and time-efficient for researchers, and effectively alleviating the possible bottleneck in the analysis pipeline. Moreover, they have shown promising results in addressing common challenges in biological experiments, such as image noise and partial image section. While these tools have substantially improved the efficiency and throughput of image processing pipelines, accuracy and methodologies continue to evolve, with ongoing development providing insight refining these technologies. Despite the advancements, the role of expert judgement and the quality of input images remain crucial. Even the most advanced algorithms require high-quality data to function optimally, and expert oversight is essential to accurately interpret the complexities of neural images. Therefore, quality control is indispensable when using these advanced tools to maintain the integrity and reliability of the results. (Table 2)

**Table 2:** Summary of selected whole-brain registration and segmentation tools

Software	Platform	Reference Atlas	Registration	Segmen-tation	Axon Tracing	Pretrain	Reference
aMAP	Open-source software	Allen Mouse Brain Atlas	3D using NiftyReg	Yes	No	No	[44]
BIRDS	ImageJ plugin	Allen Mouse Brain Atlas	3D using Elastix	Yes	No	No	[53]
D-LMBmap	Open-source software	Allen Mouse Brain Atlas	3D using Elastix	Yes	Yes	No	[51]
DeepMapi	Open-source software	Allen Mouse Brain Atlas	2D using ANTs	Yes	No	Yes	[49]
DeepSlice	Open-source software	Allen Mouse Brain Atlas	2D using QuinkNII	No	No	No	[47]
FASTMAP	ImageJ plugin	Custom, based on Allen Mouse Brain	2D using reference points	Yes	No	No	[43]
Magallen Mapper	Standalone software	Multiple	3D using Elastix	Yes	No	No	[46]
mBrainAligner	Open-source software	Multiple	3D using reference points	Yes	No	No	[50]
Mesonet	Open-source software	Allen Mouse Brain Atlas	2D using reference points	Yes	No	No	[48]
MIRACL	Standalone Software	Allen Mouse Brain Atlas	3D using ANTs	Yes	Yes	No	[45]
Neuroinfo	Standalone software	Allen Mouse Brain Atlas	3D using ANTs	Yes	No	No	[41]
WholeBrain	Standalone software	Custom, based on Allen Mouse Brain	2D using reference points	Yes	No	No	[40]

## Mapping brain connectivity through feature extraction

So far, we have discussed atlas-based registration and segmentation tools, which are indispensable for comprehensive region-to-region connectivity analysis. However, obtaining more detailed insight into individual neuronal compositions – such as the number of specific cell types or synaptic proteins – require additional steps. Researchers typically utilize high-resolution imaging of cells and specific immunostaining-labeled molecules to extract these crucial features. These image datasets require other processing steps beyond basic atlas-based registration and segmentation, although they similarly rely on feature extraction through segmentation. Firstly, despite significant advancements in imaging technologies, further image processing is essential to eliminate noise and enhance resolution, enabling accurate segmentation of somas and neurons. Neuro-reconstruction poses particular challenges due to the difficulty of extracting fine structures from often noisy images. In the following sections, we will outline image processing techniques aimed at image quality through noise reduction and resolution enhancement, followed by detail methods for cellular detection and neuron morphology reconstruction.

### Low-level image processing

Low-level image processing is essential to remove unwanted attributes and artifacts that may be misinterpreted as meaningful signals in biological image sets. Image denoising and super-resolution techniques are crucial for enhancing the quality and resolution of neural images, thereby facilitating studies in connecto-informatics. These images are often compromised with noise, artifacts, and limited resolution, which can obscure accurate interpretation and analysis. Image denoising techniques aim to reduce noise and improve the clarity of images, while super-resolution methods aim to increase the resolution and detail of low-resolution images. Together, these image processing techniques hold immense promise for advancing our understanding of the brain’s structure and function. High-quality neural images, refined through denoising and super-resolution processes, enable more accurate segmentation, precise localization of neural activity, and detailed analysis of brain connectivity.

#### Image denoising

Image denoising involves removing or reducing unwanted noise while preserving essential image features and structures in neural images. This noise can originate from various sources, including labeling imperfections, signal acquisition processes, and innate tissue features. Image denoising techniques utilize statistical models, filtering algorithms, and increasingly, machine learning approaches to effectively suppress noise and improve the image’s signal-to-noise (SNR) [54]. These techniques are particularly crucial for fluorescence images, where specific noise patterns and characteristics must be accurately managed to ensure precise data analysis and interpretation. However, the challenges of image denoising are significant, especially when the original images are of low quality. High noise levels and low resolution complicate the denoising process, making it difficult to distinguishing between noise and essential image features.

The advent of deep learning has brought significant attention to advanced image denoising algorithms. Initially, supervised learning methods like denoising CNNs were prevalent, but they require extensive high-resolution training data, which can be challenging to obtain for fluorescent biological images [55–58]. Consequently, recent developments have shifted towards self-supervised methods, which can operate with minimal or even single-image datasets.

One of the earliest deep learning-based image denoising methods that presented a solution to the difficulty in obtaining training data was CARE, which used the U-Net architecture to enhance the quality of images using pairs of low and high SNR images as training datasets [59]. More recently, frameworks like Wang et al.’s [60] use transfer learning to integrate supervised and self-supervised learning to maintain denoising performance without extensive training datasets. Noise2Void introduced a novel approach using CNNs to leverage the inherent noise characteristics within single noisy images, employing a blind-spot strategy that allows training directly on the data without needing a clean target image [61]. Despite these advancements, practical challenges remain, such as the correlation of noise among adjacent pixels in microscopy, which Noise2Void’s assumptions may not address. Structured Noise2Void [62] and Noise2SR [63] have evolved these concepts by enhancing self-supervised learning techniques and integrating super-resolution modules to improve training and denoising outcomes.

MCSC-net is another image-denoising approach tailored exclusively to fluorescent images that utilizes DNN for training and modeling the noise in the image using a Poisson-Gaussian distribution [57]. Real-time denoising methods like DeepCAD-RT uses adjacent frames for training, enabling denoising during ongoing imaging processes [64]. However, challenges such as brightness shift due to non-zero-mean noise have been addressed by innovative algorithms like ISCL: Independent Self-Cooperative Learning for Unpaired Image Denoising [65] (Fig. 1d). This method uses self-supervised learning and cyclic adversarial learning for unpaired learning and has been shown to outperform other unpaired and blind denoising methods.

The primary goal of image denoising in fluorescent imaging is to facilitate further processing, and accordingly, many algorithms are designed to incorporate additional processing steps beyond mere denoising. For instance, DenoiSeg used a self-supervised learning approach for denoising and segmentation using a single noisy image [66]. Similarly, Deconoising employs a self-supervised method that combines denoising with deconvolution of fluorescent images, allowing sharper and clearer images, essential in images with fine structures such as axons [67].

As deep learning-based image denoising continues to evolve, it remains essential for enhancing feature detection in neural imaging, crucial for analyzing neural connectivity and function. However, the application of these tools involves careful consideration of balance between reducing noise and preserving crucial image details [68]. Over-denoising may results in the loss of important details, while under-denoising may leave excessive noise, potentially leading to data misinterpretation, which makes diligent judgement from the users to maintain balance. Additionally, the generalizability of these algorithms is challenged by variability in imaging conditions and data diversity, underscoring the need for comprehensive training datasets. Despite these challenges, these algorithms significantly enhance the SNR, facilitating more accurate segmentation, visualization, and interpretation of neural structures. This improvement is indispensable for neural circuit analysis and mesoscale connectome mapping, serving as a key preprocessing step in fluorescence microscopy.

#### Image super-resolution

Super-resolution image processing techniques are crucial for enhancing spatial resolution and detail, particularly important in 3D microscopy, where axial resolution is typically two times worse than the lateral resolution, creating resolution anisotropy [69]. These techniques, using interpolation, regularization, and advanced learning-based methods, reconstruct missing details by leveraging spatial and contextual information within images [70, 71]. Reconstruction-based approaches combine multiple low-resolution images to recapture lost high-frequency components, whereas deep learning-based methods predict these components to refine image resolution [72].

Despite these advances, practical challenges persist, including high computational demands, sensitivity to input image quality, and steep learning curves, particularly for users without a background in computational imaging or machine learning. Additionally, the dependency on extensive, high-quality training datasets for learning-based methods limits their applicability across different microscopy modalities due to data availability and representativeness issues.

Addressing these problems, recent innovations have been proposed. Weigert et al. [73] introduced a super-resolution framework that reconstructs isotropic 3D data by pairing high-resolution lateral images with low-resolution axial images blurred from a non-isotropic image for training the network. Generative adversarial network (GAN)-based frameworks have been pivotal, utilizing matched pairs of low- and high-resolution images acquired through experiments for training [74]. Another GAN-based approach uses an image-degrading model to artificially create low-resolution images required for training, derived from their high-resolution counterparts, allowing the network to reconstruct super-resolution images from new low-resolution inputs [75].

In scenarios where training data is scarce, particularly in fluorescent microscopy, Eilers and Ruckebusch [76] introduced a non-deep learning super-resolution algorithm that employs interpolation on single images, requiring no training for a fast, simple resolution improvement. For cases with a limited training set available, a CNN-based approach was proposed for super-resolution [77]. Deep-SLAM, focusing on light-sheet microscopy, uses DNNs to restore z-axis resolution using raw lateral slices and degraded resolution as paired training data to restore the isotropic resolution of axial slices [78].

Particularly noteworthy are cycleGAN-based algorithm [79] and Self-Net’s [80] rapid, self-supervised learning approach, which minimize the need for extensive datasets by leveraging high-resolution lateral images as training targets for low-resolution axial counterparts. These methods streamline the training process, reduce computational requirements, and facilitate high-quality image restoration across all types of 3D fluorescence microscopy.

Super-resolution processing not only enhances image resolution beyond the limits of current imaging technology but also improves visualization of fine structures, such as neuronal components. Although there are numerous promising developments and research efforts on super-resolution algorithms, a universally applicable method for various modalities has not yet been developed. Establishing a standardized method would greatly benefit researchers, integrating these advancements into the connecto-informatics image processing pipeline.

### Cell segmentation

The brain comprises a multitude of cell types, such as neurons and glial cells, ditinguished by their morphology, topographic position, molecular signatures and so forth. Cell segementaion (i.e. cell body or soma) provide information about cell density and type in distinct brain regions that is crucial for understanding the intricate organization of brain connectivity at the cellular level. Variations in these attributes, like cell density and type, within specific brain regions have been linked to neurological disorders such as Parkinson’s disease [81–86]. The 3D topographical organization of cells, which relates to cell-type-specific connectivity, further highlights the complexity of neural networks [10]. Techniques like STPT have enabled researchers to map spatial cell type distributions withn the cerebrovascular network, revealing the elaborate cellular organization underlying brain circuits [87, 88]. Accurate detection and identification of cells are essential for unraveling the complexities of neural circuit connectivity, function, and organization. This understanding is pivotal for advancing our knowledge of brain functionality in both health and disease, potentially leading to improved treatments for neurological conditions.

However, accurate detection and identification of cells pose significant challenges, including the resolution limitations of current imaging technologies and the difficulty of distinguishing between cell types in densely packed regions. These issues underscore the need for advanced segmentation and identification tools for precise analysis. ImageJ, a conventional image analysis tool, facilitates soma detection through plugins that allows segmentation and quantification via manual parameter adjustments [89, 90]. Yet, the rapid advancement in imaging technology has produced large-scale, high-resolution images, making manual segmentation time-consuming and labor-intensive.

To address this, several automatic 3D soma detection algorithms for fluorescent images have been developed [91–93]. One algorithm focused on automatic large-scale 3D soma detection through multiscale-morphological closing and adaptive thresholds implemented to the images [94]. The shift from manual manipulation to automated algorithms marks a significant development in cellular-level analysis in neural circuit mapping. Continuous efforts are being made to overcome the almost impossible manual-intensive cellular segmentation, and recently, AI has been implemented to overcome the issue [95]. Particularly, deep learning-based approaches have been instrumental in advancing cell detection [96]. CNNs have been trained to detect and segment densely packed cells automatically, even in partially labeled datasets, revealing crucial topographical information and into possible cell-type-specific functions about PV cells in the STN [10, 97] (Fig. 1e). Another method uses DNN to automatically detect 3D soma in a mouse whole-brain image, which allows the detection of a large population of cells [98].

Tools like Fiji/ImageJ, enhanced with deep learning plugins such as DeepImageJ, allow users to either use pre-trained CNN models or train their models for cell detection tasks [99]. Despite the advantages of deep learning-based methods, they often face challenges such as slow processing due to the need for extensive data training, limitations in applying to whole-brain images, or handling high-throughput image generation. Recent methods like a two-stage DNN-based algorithm have been developed for fast and accurate soma detection in whole mouse brain images, addressing these challenges by filtering images without somas and segmenting those with identified somas [100].

Further advancements include weak- and self-supervised cell segmentation methods developed to reduce the burden of manually making pixel-level ground-truth training labels [101, 102]. Open-source software like Cellpose, which uses a U-Net-based algorithm, requires minimal user intervention and allows rooms for additional training, making it accessible and user-friendly for various cell segmentation tasks [103].

While accurate cell segmentation is crucial for further brain mapping analysis at the cellular level, further quantification and identification are also essential in following connecto-informatics analysis. CellProfiler [104], an early software widely used for cell phenotype identification, and newer tools such as CellCognition and CellSighter, use deep learn and unsupervised learning to automate the analysis of cell based on their phenotypes [105, 106]. Another algorithm demonstrated the accurate classification of cells by their phenotypes in a mixed cell population image with high accuracy [107]. This algorithm used self-label clustering, where the primary objective was to achieve precise cell identification based on morphological characteristics. These tools offer potential for expedited circuit mapping analysis, alleviating a time-consuming bottleneck in the workflow.

Accurate cell detection and identification are pivotal for exploring the morphological, connectivity, and functional aspects of cells, thereby enhancing our understanding of mesoscale neural circuits. Although automated and manual detection methods offered by various software tools facilitate this analysis, challenges such as high variability in cell morphology and potential algorithmic bias in automated tools can affect the reliability of cell identification and subsequent analyses. Recognizing and addressing these challenges is essential for advancing our comprehension of neural circuitry, function, and organization.

### Neuronal morphology reconstruction

Neurites are cellular processes that project from the cell body of a neuron. These extensions encompass both axons and dendrites, essential for neural communication and connectivity, facilitating information transmission throughout the nervous system. Digitally reconstructing these neuronal morphologies from imaging data enables the analysis and integration of neural networks across various modalities. Recent advancements in computer-assisted tracing algorithms and technologies have enabled large-scale neuron reconstruction efforts, providing insights into the brain’s mesoscale connectivity patterns and enhancing our understanding of its structure and organization [108, 109]. However, challenges such as high computational cost and the technical complexity of capturing detailed neuronal structures persists, highlighting the need for advances tools in neuron reconstruction.

NeuTube1.0, an open-source platform, allows for detailed neuron reconstruction neural tracing [110]. It facilitates both 2D and 3D visualization and tracing of neurons for reconstruction from fluorescent images, employing a semi-automatic approach, with seed-based tracing and path-searching algorithms within a cylindrical fitting model. This method allows efficient visualization, reconstruction, and editing of neuron structures, providing a valuable resource for researchers (Fig. 1f). Through neuTube1.0, researchers have analyzed the spatial synaptic connectivity pattern of the hippocampus region using mGRASP and shown in reconstructed 3D neuron structures [12, 111]. Additionally, neuTube1.0 was used to create a comprehensive atlas of the larval zebrafish brain at cellular resolution by systematically mapping the cellular composition and connectivity patterns of 1,955 reconstructed single neurons [112].

Another open-source program, Vaa3D, integrated with TeraFly and TeraVR, is a cross-platform visualization and analysis system that allows visualization on tera-byte scale images and neuron tracing in a virtual-reality environment [113, 114]. TeraFly efficiently handles large-scale 3D image data, focusing on specific regions of interest with varying levels of details, while TeraVR provides an immersive environment for neuron reconstruction, facilitating precise tracing and annotation [115, 116]. Utilizing the ‘Virtual Finger’ algorithm, Vaa3D has facilitated the semi-automatic tracing of over 1,700 neurons from mouse brain images obtained using the fMOST, revealing the morphological diversity of single neurons in a brain-wide scale [117]. Additionally, the same tools were used to characterize neurons in the human brain by reconstructing 852 neurons from images obtained using a newly proposed cell adaptive tomography (ACTomography) to capture cortical neurons injected with dyes that targeted individual neurons in the human brain tissues [118].

The MouseLight project has reconstructed the morphology of 1,000 projection neurons using a semi-automatic pipeline that classifies axonal structures, generates a probability stack for skeleton extraction and segmentation, and refines axonal segment reconstructions through human annotation [119]. This project has uncovered previously unknown cell types and elucidated the organization of long-range connections within the mouse brain.

Recent work in cortical cell subtype mapping has reconstructed 6,357 single neurons in the mPFC through the Fast Neurite Tracer (FNT) software tool using images obtained with fMOST, classify axon projections into subtypes and revealing the topographical organization of PFC axon projections [120]. The FNT software facilitates the tracing of large image datasets by dividing them into smaller three-dimensional cubes. It employs Dijkstra’s algorithm, a method for finding the shortest paths between nodes in a graph, which in this context, helps visualize and trace neurons accurately by determining the most efficient routes for neuron paths. Furthermore, using the single-neuron reconstruction data traced through neuTube and FNT, Gao et al. [121] further reconstructed over 2,000 neurons and classified the organization into finer subtypes based on the axon-dendrite features, which revealed inter-connectivity among projection neuron types in the PFC. Most recently, Qui et al. [122] reconstructed 10,100 single neurons to map the brain-wide spatial organization of neurons in the mouse hippocampus. By manually reconstructing single neurons, they revealed patterns and subtypes of neurons within the hippocampus, which serve as a basis for understanding its functions further.

Although semi-automatic tools predominate, significant strides have been made in developing automatic algorithms for neuron reconstruction [123–126]. Yet, the inherent variability in datasets, influenced by different animal models, imaging techniques, and neuron types, presents considerable challenges to solely rely on automatic algorithms [127–131]. Automated algorithms also face challenges with densely interwoven dendrites and axons from multiple labeled neurons.

While existing methods excel in single neuron morphology, they struggle with accurately reconstructing densely structured neurons. Computational strategies like the TREES toolbox simulate and analyze the complex branching patterns of neurons based on branch order to reconstruct multiple neurons, while NeuroGPS-Tree utilizes spatial information of cell bodies and statistical distribution to iteratively detect and eliminate incorrect connections between two neuron reconstructions to accurately separate intertwined neurons [132, 133]. Li et al. [134] introduced G-Cut, a novel development that segments densely interwoven neuron clusters. This tool uses a graph-based representation of cell bodies to calculate the global optimum, automatically segmenting individual neurons within a cluster. G-Cut demonstrates higher accuracy in segmentation compared to previously mentioned methods. GTree was developed as an open-source tool for brain-wide dense neuron reconstruction by building on NeuroGPS to identify neurons and integrate a display module to check errors for higher reconstruction accuracy [135].

While software tools like NeuroGPS, TREES toolbox, and G-Cut advance neuron reconstruction, they often overlook errors such as neuron entanglement and interference from passing axons, which are crucial for pruning. The SNAP pipeline addresses this gap by offering structured pruning to eliminate reconstruction errors and disentangle neuron reconstructions, enhancing accuracy and reducing the need for manual curation [136].

Despite these state-of-the-art advances, semi-automatic methods are preferred in large-scale brain-wide neuron reconstruction efforts. Central to understanding the limitations and potentials of automated tracing algorithms is the BigNeuron project, a collaborative project aimed at benchmarking the performance of these algorithms across diverse light microscopy datasets [137]. BigNeuron aims to enhance automatic neuron tracing tools by offering a standardized comparison platform. It creates a diverse, cross-species dataset for benchmarking, provides gold standard annotations for select datasets, and evaluates 35 automatic tracing algorithms. This initiative advances algorithm development for broader benchmarking and underscores the importance of human expertise in generating gold-standard datasets for accurate comparisons.

The evolution of AI, particularly deep learning, offers a promising future for neuron reconstruction, automating tasks that once heavily relied on human expertise, especially in dataset preparation. Emerging methods are significantly reducing, and in some cases eliminating, the need for human intervention in creating training datasets for neuron reconstruction. By combining traditional tracing methods to create pseudo-labels needed for training and the 3D deep learning network for neuron reconstruction, Zhao et al. [138] suggested a neuron tracing framework that does not require manual annotation. Another novel approach utilized a weakly supervised CNN for a fully automatic neuron tracing method, including generating automatic training labels [139]. This method was further improved to detect and trace distorted or broken structures using probability maps estimated by 3D residual CNN [140].

Additionally, using a self-supervised approach, a 3D CNN was used to predict the order of permuted slices in the 3D image, leveraging the tube-like structure of axons for label-free feature extraction and enhancing downstream segmentation with a 3D U-Net model [141]. MPGAN also utilized a self-supervised method to develop a two-stage generative model strategy that creates synthetic 3D images with voxel-level labels from unlabeled data, enhancing segmentation network performance and improving neuron reconstruction methods [142]. These approaches promise to alleviate the bottleneck in neuron tracing by streamlining the process of generating training datasets and applications.

Neuron reconstruction is critical for analyzing neural circuits, including measurements like dendritic length and synaptic connections. Incorporating deep learning into this process marks a significant shift towards automation, reducing the dependence on human expertise. Future improvements should focus on enhancing the models’ accuracy, reliability, and generalizability. As deep learning evolves, it offers biologists advanced tools for uncovering the complex organization of neural structures. However, challenges related to data quality, algorithmic adaptability, and the integration of diverse imaging data remain, highlighting the need for continued innovation in automated neuron reconstruction methodologies (Table 3).

**Table 3:** Summary of selected cell and neuron reconstruction tools

Software	Platform	Language	Function	Learning-based	Method	Reference
CellCognition	Open-source software	Python	Cell classifier	Yes	CNN	[105]
Cellpose	Open-source software	Python	Cell segmentation	Yes	U-Net	[103]
CellProfiler	Open-source software	MATLAB/C++	Cell classifier	No	-	[104]
CellSighter	Open-source software	Python	Cell classifier	Yes	CNN	[106]
DeepImageJ	Fiji plugin	Java	Cell segmentation	Yes	CNN	[99]
APP2	Vaa3d plugin	C++	Neuron reconstruction	No	-	[108]
FNT	Standalone software	C++	Neuron reconstruction	No	-	[120]
G-Cut	Open-source software	Matlab	Neuron reconstruction	No	-	[134]
Huang et al.	Open-source software	Python	Neuron reconstruction	Yes	CNN	[139]
Klinghoffer et al.	Open-source software	Python	Neuron reconstruction	Yes	3D U-Net	[141]
MPGAN	Open-source software	Python	Neuron reconstruction	Yes	GAN	[142]
NeuroGPS	Vaa3d plugin	C++	Neuron reconstruction	No	-	[133]
neuTube1.0	Standalone software	C++	Neuron reconstruction	No	-	[110]
TeraVR	Vaa3d plugin	C++	Neuron reconstruction	No	-	[116]

## Discussion & conclusion

In summary, this review provides an up-to-date overview of the current advances in image processing tools, highlighting the integration of AI to tackle the challenges arising from the growing volume and diversity of generated images. The integration of AI has shown promising results in alleviating the image processing bottleneck, potentially revolutionizing the field. However, the need for manual intervention persists due to factors such as quality variants and complexity in neural data. Additionally, certain advanced tools may initially encounter accessibility limitations or implementation constraints across different modalities.

While AI framework may provide enhanced accuracy and faster image processing speed, the inherent features found in neural data makes human intervention inevitable. Moreover, the challenge of gathering sufficient training datasets for deep learning poses as a significant limitation. Ongoing efforts are being made to overcome these challenges, aiming to integrate deep learning throughout the image processing workflow more comprehensively. This integration aims to minimize manual input and provide a more unified, efficient image processing pipeline that accommodates various experimental and imaging approaches. Such an approach is crucial for expedited analysis of mesoscale brain connectivity mapping data, highlighting continuous pursuit towards automation while acknowledging the indispensable role of human expertise.

Connecto-informatics, as applied at this level of analysis, holds great promise in illuminating the underlying mechanisms behind diverse brain functions and the development of neurological diseases linked to disruptions in neural circuits. Furthermore, it is essential to note that advanced tools for connecto-informatics at the microscale are equally significant despite being omitted in this review. As the field continues to evolve, the pivotal role of interdisciplinary collaboration and the integration of cutting-edge technologies cannot be overstated. These collaborative efforts will undoubtedly drive further advancements in our comprehension of brain connectivity at the mesoscale level, paving the way for new insights and potential therapeutic strategies.

## Data Availability

No datasets were generated or analysed during the current study.
